# Direct Comparison of Two Commercially Available Pulsed Field Ablation Systems for Atrial Fibrillation; Procedure Characteristics and Acute Outcomes

**DOI:** 10.1111/jce.16761

**Published:** 2025-06-12

**Authors:** Bob G. S. Abeln, Marisa van der Graaf, Dan L. Musat, Melanie A. Gunawardene, Devi G. Nair, Bradley P. Knight, Bradley Wilsmore, Max Liebregts, Vincent F. van Dijk, Maurits C. E. F. Wijffels, Jippe C. Balt, Suneet Mittal, Stephan Willems, Nishant Verma, Graham Peigh, Michael M. Malaty, Lucas V. A. Boersma

**Affiliations:** ^1^ Department of Cardiology St. Antonius Hospital Nieuwegein the Netherlands; ^2^ Department of Cardiology Amsterdam UMC Amsterdam the Netherlands; ^3^ Snyder Center for Comprehensive Atrial Fibrillation, Valley Health System Paramus New Jersey USA; ^4^ Department of Cardiology and Intensive Care medicine Asklepios St. Georg Clinic Hamburg Germany; ^5^ Department of Cardiac Electrophysiology St. Bernards Medical Center Jonesboro Arkansas USA; ^6^ Division of Cardiology Northwestern University, Feinberg School of Medicine Chicago Illinois USA; ^7^ Department of Cardiology John Hunter Hospital New Lambton New South Wales Australia

**Keywords:** atrial fibrillation, catheter ablation, efficacy, pulmonary vein isolation, pulsed field ablation, safety

## Abstract

**Background:**

There are few comparative studies on the everyday clinical outcomes of commercially available pulsed field ablation (PFA) systems for atrial fibrillation (AF).

**Objective:**

This study evaluates the acute efficacy and safety outcomes of the FARAPULSE™ (pentaspline catheter) and PulseSelect™ (circular catheter) system.

**Methods:**

International, multicenter, registry on patients with paroxysmal or persistent AF, undergoing a first ablation using either PFA system between January 29th, 2024 and September 1st, 2024. Primary endpoints were electrical isolation of ablation targets, procedural characteristics and freedom from major adverse events within 30 days postprocedure.

**Results:**

A total of 402 patients were enrolled at the six participating centers, of whom 56.5% were treated with the pentaspline ablation‐catheter. Acute procedural efficacy was 100% for both groups. Use of the pentaspline ablation‐catheter was associated with significantly shorter procedural times (36.0 min [IQR 31.0; 44.0] *vs.* 49.0 min [IQR 41.5;76.0]) compared to the circular ablation‐catheter (*p* < 0.001). Major adverse events were scarce and not different between cohorts. Two patients (0.5%) experienced a stroke and one patient (0.2%) had a serious vascular access site complication. One patient had a transient ischemic attack (0.2%). Minor vascular access site complications were more common in the pentaspline catheter group (11.9% vs. 1.1%, *p* < 0.001).

**Conclusions:**

This study showed excellent acute efficacy and safety for both PFA‐systems. Our findings reveal shorter procedural times with the pentaspline catheter, less minor access site complications with the circular catheter, and several procedural differences between the ablation systems, often driven by operator choice.

## Graphical abstract

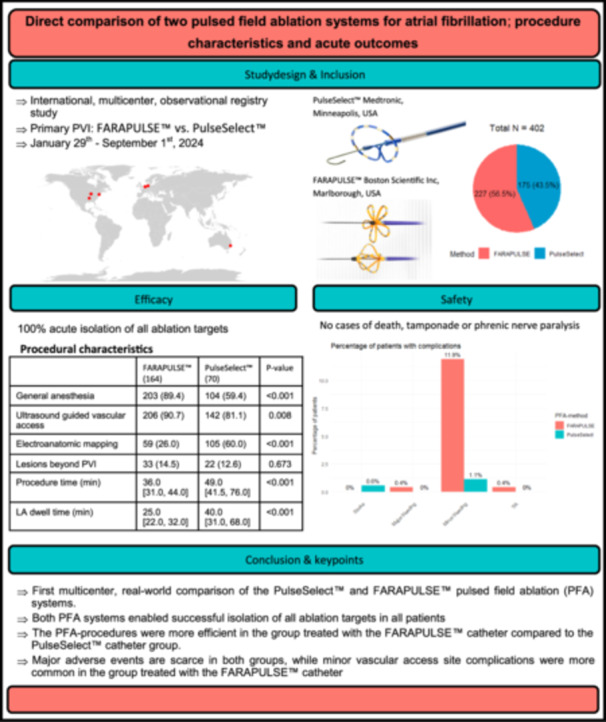

**Abbreviations**
AF: atrial fibrillation
ICE: intracardiac echocardiography
LAVI: Left Atrial Volume Index
PFA: pulsed field ablation
PV: pulmonary vein
PVI: pulmonary vein isolation
TEE: transesophageal echocardiogram
TIA: transient ischemic attack

## 1 Introduction

Pulsed field ablation (PFA) has emerged as a novel ablation modality for catheter ablation of cardiac arrhythmias. In contrast to radiofrequency‐ and cryoablation, that use thermal energy to induce cell necrosis, PFA systems create lesions by applying multiple short (milli‐ to nanosecond), high voltage electrical pulses to the myocardium [1, 2]. These pulses create nanopores in the cell membrane that induce apoptosis leading to transmural lesions while potentially sparing noncardiac tissue and avoiding complications such as phrenic nerve injury, pulmonary vein stenosis, and atrioesophageal fistula [3, 4, 5, 6, 7]. Additionally, PFA is faster compared to thermal ablation, which appears to shorten procedure times and improve procedural efficiency [8].

Although various PFA systems have been introduced recently, relatively little is known about the differences in procedural safety and efficacy among these systems. Currently, there are four PFA systems that are commercially available in both Europe and the USA: the FARAPULSE™ system (Boston Scientific, Marlborough, USA), characterized by a pentaspline ablation catheter; the PulseSelect™ system (Medtronic, Minneapolis, USA) utilizing an over‐the‐wire circular ablation catheter, the VARIPULSE™ system (Biosense‐Webster, Irvine, USA), that is characterized by a circular ablation catheter that is enhanced for electroanatomic mapping, and the Affera™ system (Medtronic, Minneapolis, USA), which utilizes a lattice tip mapping and ablation catheter. Approval for clinical use of these systems has been established by adequate safety and efficacy in premarket studies [9, 10, 11, 12]. Aside from the FARAPULSE™ system, limited data is available on everyday clinical practice outcomes of other PFA systems [3, 13, 14, 15]. Furthermore, a direct comparison of different PFA systems has not yet been performed.

In this study, we evaluated the acute procedural outcomes of two of the currently commercially available PFA systems. The acute procedural safety and efficacy were assessed in a real‐world clinical setting, enabling a head‐to‐head comparison of those two PFA systems for catheter ablation of AF.

## 2 Methods

### 2.1 Trial Design

This was an international, multicenter, observational registry study that aimed to evaluate the acute procedural safety and efficacy of the FARAPULSE™ (Boston Scientific Inc., MA, USA) and PulseSelect™ (Medtronic, MN, USA) pulsed field ablation systems. At each center, the local ethics committee approved the collection of the required data and the study was performed in accordance with the Declaration of Helsinki.

### 2.2 Study Participants

The study was performed in patients treated between January 29th and September 1st 2024 following the commercial launch of PulseSelect™ in Europe, and the commercial launch of PulseSelect™ and FARAPULSE™ in the United States. European operators therefore had prior commercial experience with FARAPULSE™ while US based operators did not. Patients with AF undergoing a first pulmonary vein isolation (PVI), and treated with PFA ablation were consecutively included in the study. Patients with a prior left atrial ablation procedure were excluded from the study. Patients were prospectively entered in each center's local ablation database and then retrospective collection and analysis was performed by merging the relevant data from all six participating centers (Asklepios St. Georg Clinic (Germany), John Hunter Hospital (Australia), Northwestern Memorial Hospital (USA), St. Antonius Hospital (The Netherlands), St. Bernards Medical Center (USA) and Valley Health System (USA)).

### 2.3 Procedure

The ablation procedures were performed per local institutional standards (see Table 1) in accordance with the instructions for use of the ablation systems. Procedures could be performed under general anesthesia or conscious sedation. Typically, oral anticoagulation therapy was uninterrupted or paused in the morning of the procedure, while antiarrhythmic drugs were continued at the discretion of the operator. Vascular access was obtained with or without ultrasound‐guided puncture of the femoral vein. Subsequently, a diagnostic catheter was placed in the coronary sinus. Left atrial access was gained using transseptal puncture that was guided by fluoroscopy, transesophageal, or intracardiac echocardiography as per each center's standard of care. Intravenous heparin was administered either before or after transseptal puncture to maintain a therapeutic ACT. Atropine or glycopyrrolate was administered at the operator's discretion to prevent a vagal response. Assessment of left atrial and pulmonary vein (PV) anatomy was performed using fluoroscopy or an electroanatomic mapping system (EnSite™, Abbott Cardiovascular, Plymouth, USA, or CARTO™, Biosense Webster, Diamond Bar, USA) as deemed appropriate by the operator. Subsequently all pulmonary veins were targeted with the PFA system, using the typical settings for the different systems (see Table 2). At the discretion of the treating physician, additional structures (e.g. left atrial posterior wall) could be targeted with the PFA system. At the end of the procedure, the effect of the ablation was assessed by checking for entrance and/or exit block, and additional electroanatomic mapping was performed at the operator's discretion. In case an ablation target was not successfully treated, additional PFA applications were allowed. After the final energy application and assessment of the desired endpoint, the catheters and sheaths were removed from the patient and hemostasis was achieved using manual pressure and a figure of eight suture or with a vascular closure device. The timing of hospital discharge was determined by institutional standards, typically the same day or the day after the ablation procedure.

**Table 1:** Institutional standards.

	St. Antonius hospital	Valley health system	Asklepios St. Georg clinic	St. Bernards medical center	Northwestern memorial hospital	John hunter hospital
General anesthesia	92%	27%	5%	100%	100%	73%
Single or two operator(s) per procedure	Single operator	One or two operators	Single operator	Single Operator	Single operator plus trainee	Single operator
Ultrasound guided vascular access	77%	100%	100%	100%	100%	100%
Use of ICE periprocedural	0%	100%	0%	100%	100%	0%
Use of TEE periprocedural	0.9%	0%	58%	0%	0%	0%
Electroanatomic mapping system	3%	93%	93%	100%	100%	56%
Lesions beyond PVI	SVC: 0.4%	Posterior wall: 36.0%	Posterior wall: 2.3%	SVC: 30.6%	Posterior wall: 50.0% MI‐line: 2.9% CTI‐line: 2.9% Right atrial flutter ablation: 2.9%.	Posterior wall: 63.6% MI‐line: 9.1%
Vascular closure	Figure of 8 suture and manual pressure	Closure device	Figure of 8 suture and manual pressure	Closure device	Figure of 8 suture and manual pressure	Closure device
Protamine	35%	7%	0%	100%	35%	9%

*Note:* Numbers in the cells are percentages of the total number of patients treated in that center.

Abbreviations: CTI‐line, cavotricuspid isthmus line; ICE, intracardiac echocardiography; MI‐line, mitral isthmus line; SVC, superior vena cava; TEE, transesophageal echocardiogram.

**Table 2:** Ablation system characteristics.

	FARAPULSE™	PulseSelect™
Catheter design	Pentaspline, 20 electrodes, 31 or 35 mm diameter	Circular, nine electrodes, 25 mm diameter
Catheter configurations	Linear, basket, or flower	Circular
Catheter shaft	12 F diameter, 115 cm usable length, guidewire compatible	9 F diameter, 99 cm usable length, guidewire compatible
Access sheath	FARADRIVE™ Steerable Sheath, 16.8 F outer diameter	FlexCath Contour™ Steerable Sheath, 14.0 F outer diameter
Pulse profile	Pulsetrain of five biphasic, bipolar pulses at 2 kV, unsynchronized to the cardiac cycle	Pulsetrain of four biphasic, bipolar pulses at 1.4–1.5 kV, synchronized to the cardiac cycle
Typical applications per PV	Four basket in two overlapping positions, and four flower applications in two overlapping positions	Four ostial in four 90‐degree rotated positions and four antral in four 90 degree rotated positions

### 2.4 Endpoints

The primary efficacy endpoint was acute procedural success, defined as the achievement of electrical isolation of all ablation targets at the end of the ablation procedure. Secondary efficacy endpoints included the number of PFA applications per ablation target, total procedure time (time from first venous access to last sheath removal), left atrial dwell time (time from transseptal puncture to removal of the last catheter from the left atrium), fluoroscopy duration, and dose. The primary safety endpoint was freedom from major adverse events during or within 30 days after the ablation procedure, including cardiac tamponade, stroke, systemic thromboembolism, persistent phrenic nerve injury, vascular access site complications requiring surgical intervention or transfusion, and death. Secondary safety outcomes included minor adverse events, including TIA, vagal reactions during the procedure, minor vascular access site complications (not requiring surgical intervention or transfusion) and transient phrenic nerve injury. Finally, sub analyses were performed to assess the relation between procedural characteristics and efficacy and safety outcomes (Supporting Information Tables 1, 2, and 3).

### 2.5 Data Analysis and Statistics

Data analysis was conducted at the St. Antonius Hospital using R, version 4.4.0 (R Foundation, Vienna, Austria). The statistical methods used were descriptives, as this was an observational study which was not driven by a formal endpoint hypothesis. Baseline and procedure variables were presented as mean ± SD or median [IQR] for continuous variables, and frequencies with percentages for categorical variables. Comparisons between groups were performed using students t‐test for normally distributed continuous data, Mann‐Whitney U test for non‐normally distributed continuous data, and *χ* ^2^ test for categorical data. A significance level α of 0.05 was used. Missing data were not imputed.

## 3 Results

A total of 402 patients were enrolled between January 2024 and September 2024 at the six participating centers. The pentaspline ablation catheter was used in 227 (56.5%) patients and the remainder were treated with the circular ablation catheter.

### 3.1 Baseline Characteristics

Baseline characteristics of the patients, stratified per ablation system are presented in Table 3. In brief, the cohort consisted of mostly males (66.4%) with a mean age of 62.9 ± 10.5 years and left atrial enlargement (LAVI > 35 mL/m2) being present in 32.4% of the cohort. Most baseline characteristics were comparable between the two treatment arms. Notably, class I or III antiarrhythmic drugs were used significantly more often in the group that was treated with the pentaspline ablation catheter (64.8% *vs.* 48.0%, *p* < 0.001). In contrast beta‐blockers were prescribed significantly more often in the circular ablation catheter group (53.1% *vs.* 39.6%, *p* = 0.01).

**Table 3:** Baseline characteristics.

	FARAPULSE™ (227)	PulseSelect™ (175)	*p* value
Female sex	77 (33.9)	58 (33.1)	0.954
Age, years	62.0 ± 10.7	64.1 ± 10.1	0.050
BMI, kg/m^2^	28.0 ± 5.8	28.2 ± 4.8	0.708
Hypertension	92 (40.5)	71 (40.6)	1.000
Diabetes mellitus	23 (10.1)	22 (12.6)	0.542
Heart failure	26 (11.5)	25 (14.3)	0.487
Coronary artery disease	19 (8.4)	21 (12.0)	0.300
Chronic kidney disease (eGFR< 45)	7 (3.4)	9 (6.0)	0.358
OSAS	30 (13.2)	31 (17.7)	0.269
CHA2DS2‐VASc score			0.351
0	61 (26.9)	38 (21.7)	
1	52 (22.9)	37 (21.1)	
2+	114 (50.2)	100 (57.1)	
LAVI, mL/m2	32.0 ± 10.1	32.4 ± 9.6	0.724
Time since AF diagnosis, years	1.5 [0.6, 4.7]	1.5 [0.5, 4.5]	0.475
Type of AF			0.586
Paroxysmal AF	132 (58.1)	107 (61.1)	
Persistent AF	93 (41.0)	65 (37.1)	
Longstanding persistent AF	2 (0.9)	3 (1.7)	
EHRA symptom classification			0.504
Class 1 (No)	9 (4.5)	7 (4.5)	
Class 2 (Mild)	159 (79.5)	125 (80.6)	
Class 3 (Severe)	29 (14.5)	23 (14.8)	
Class 4 (Disabling)	3 (1.5)	0 (0.0)	
Class I or III antiarrhythmic drugs	147 (64.8)	84 (48.0)	0.001
Anticoagulant drugs	224 (98.7)	166 (94.9)	0.053

*Note:* Data is presented as counts (percentages) for categorical data, means ± standard deviations for normally distributed continuous data and median [interquartile range] non‐normally distributed continuous data.

Abbreviations: AF, atrial fibrillation; LAVI, Left Atrial Volume Index; OSAS, obstructive sleep apnea.

### 3.2 Procedural Characteristics

The procedural characteristics are presented in Table 4. Most cases were performed under general anesthesia (76.4%) and ultrasound to guide femoral vein access in 86.6%. Targets outside the PV's were targeted in 13.5% of patients. Electroanatomic mapping systems were used in a small subset of patients (40.8%). Differences in procedural characteristics between the two PFA systems were: general anesthesia was used significantly less often in patients treated with the circular ablation catheter (59.4%), compared with the pentaspline ablation catheter group (89.4%, *p* < 0.001). Likewise, ultrasound guidance for femoral vein access was also used significantly less often in the circular ablation catheter group (81.1% vs. 90.7%, *p* = 0.008). Contrastingly, echocardiographic guidance (TEE or ICE) was used more often in the circular ablation catheter group (TEE: 13.1% vs. 1.8%, *p* < 0.001 and ICE: 37.1% vs. 21.6%, *p* = 0.001). The same applies to the higher use of mapping systems in the circular ablation catheter group (60.0% vs. 26.0%, *p* < 0.001). Ablation of non‐PV ablation targets did not differ between ablation systems and included the posterior wall in the majority of cases.

**Table 4:** Procedural characteristics.

	FARAPULSE™ (227)	PulseSelect™ (175)	*p* value
General anesthesia (instead of conscious sedation)	203 (89.4)	104 (59.4)	< 0.001
Ultrasound guided vascular access	206 (90.7)	142 (81.1)	0.008
Use of echocardiography – TEE	4 (1.8)	23 (13.1)	< 0.001
Use of echocardiography ‐ ICE	49 (21.6)	65 (37.1)	0.001
Electroanatomic mapping system	59 (26.0)	105 (60.0)	< 0.001
Lesions beyond PVI	33 (14.5)	22 (12.6)	0.673
Electrocardioversion during procedure	72 (31.7)	56 (32.0)	1.000
Vascular closure device (instead of figure of eight suture)	57 (25.1)	34 (19.4)	0.219
Protamine	90 (39.6)	43 (24.6)	0.002
Procedure time (min)	36.0 [31.0, 44.0]	49.0 [41.5, 76.0]	< 0.001
LA dwell time (min)	25.0 [22.0, 32.0]	40.0 [31.0, 68.0]	< 0.001
DAP (mGy*cm^2^)	5.0 [3.0, 9.0]	6.0 [3.0, 10.0]	0.300
Fluoroscopy duration (min)	10.0 [8.0, 13.0]	11.0 [6.0, 16.0]	0.082

*Note:* Data is presented as counts (percentages) for categorical data, means ± standard deviations for normally distributed continuous data and median [interquartile range] non‐normally distributed continuous data.

Abbreviations: DAP, dose area product, ICE, intracardiac echocardiography, LA, left atrial, PVs, pulmonary veins, TEE, transesophageal echocardiogram.

### 3.3 Efficacy Outcomes

Acute procedural success, defined as the successful isolation of all ablation targets, was achieved in all patients. This was achieved with a median number of eight PFA applications per vein with both systems. There was a higher median total number of applications for the PVs in the pentaspline ablation catheter group of 34 [IQR 32; 40] compared to 33 [IQR 32; 36] applications in the circular ablation catheter group (*p* = 0.001). Additional PV applications (on top of the standard eight applications) were applied in 67.6% of patients in the circular ablation catheter group and 60.4% of the pentaspline catheter group (p = n.s.). Structures outside the pulmonary veins were targeted in 55 patients (33 patients (14.5%) in the pentaspline group vs. 22 patients (12.6%) in the circular ablation catheter group, p = n.s.) and included the posterior wall (41 patients), the lateral mitral isthmus (2 patients), superior vena cava (12 patients) and cavotricuspid isthmus (1 patient).

The median procedure duration was significantly longer in the group that was treated with the circular ablation catheter: 49.0 min [IQR 41.5; 76.0] versus 36.0 min [IQR 31.0; 44.0] in the group treated with the pentaspline ablation catheter (*p* < 0.001). In a subanalysis of patients undergoing PVI‐only without electroanatomic mapping (Pentaspline catheter group, *n* = 163; circular catheter group, n = 70), this difference persisted, with median procedure times of 34.0 min [31.0; 39.0] versus 42.0 min [36.0; 48.0], *p* < 0.001. In line with the total procedure duration, the left atrial dwell time was also significantly longer in the group that was treated with the circular ablation catheter compared to the pentaspline ablation catheter, 40.0 min [IQR 31.0; 68.0] versus 25.0 min [IQR 22.0; 32.0], respectively (*p* < 0.001). Fluoroscopy times and dose area product (DAP) were comparable between PFA systems.

Both total procedure time and fluoroscopy time were significantly affected by several procedural aspects, such as anesthesia type, echocardiography use and use of a 3D‐mapping device. These interactions are summarized in Supporting Table 1 and 2. For example, the use of echocardiography guidance with ICE was associated with significantly longer total procedure times (77.0 [IQR 45.3; 126.8] min vs 39.0 [33.0; 46.0] min, *p* < 0.001).

### 3.4 Safety Outcomes

Major adverse events during 30 day follow‐up occurred in 1 patient (0.6%) of the circular ablation catheter group and 1 patient (0.4%) of the pentaspline catheter group (Table 5). One patient experienced a major bleeding complication at the femoral access site with the presence of a pseudoaneurysm in the group treated with pentaspline ablation catheter. After injection of thrombin, the bleeding continued necessitating an intervention to place a covered stent.

**Table 5:** Periprocedural complications.

	FARAPULSE™ (227)	PulseSelect™ (175)	*p* value
Death	0 (0.0)	0 (0.0)	NA
Tamponade	0 (0.0)	0 (0.0)	NA
Thromboembolic events	1 (0.4)	2 (1.1)	0.128
TIA	1 (0.4)	0 (0.0)	1.000
Stroke	0 (0.0)	1 (0.6)	1.000
Systemic embolic event	0 (0.0)	0 (0.0)	NA
Phrenic nerve paralysis (persistent or transient)	0 (0.0)	0 (0.0)	NA
Vascular access site complications	28 (12.3)	2 (1.1)	< 0.001
Major bleeding	1 (0.4)	0 (0.0)	1.000
Minor bleeding ‐ Hb‐loss > 1 mmol/L	2 (0.9)	1 (0.6)	0.807
Minor bleeding ‐ Prolonged hospitalization > 4 h	12 (5.3)	1 (0.6)	0.008
Minor bleeding ‐ Prolonged hospitalization < 4 h	13 (5.7)	0 (0.0)	NA
Vagal reaction during ablation requiring atropine and/or temporary pacing	5 (2.2)	0 (0.0)	NA

*Note:* Data is presented as counts (percentages) for categorical data.

Abbreviations: Hb, hemoglobin; TIA, transient ischemic attack.

One patient in the circular ablation group experienced a stroke postprocedure. This patient underwent a PVI‐only procedure, had a CHA_2_DS_2_VASc score of 6 and was treated with apixaban (2.5 mg bidaily, adjusted to age and renal function), which was interrupted for 24 h before the procedure. The patient presented at the emergency department after 3 days with a headache, facial numbness, and falling. Magnetic Resonance Imaging showed signs of two small infarcted areas in the gray‐white junctions of the parietal lobe. The patient had complete resolution of symptoms. Notably, none of the patients experienced pericardial effusion or tamponade following the procedure.

Minor adverse events observed at the 30‐day follow‐up included mostly continued vascular access site bleeding (*n* = 29), predominantly affecting patients in the pentaspline catheter group (Pentaspline catheter: *n* = 27, 11.9% vs. Circular catheter: *n* = 2, 1.1%, *p* < 0.001). In 16 patients (55.2%) the hospital stay was extended with at least 4 h for this reason. Furthermore, three of these patients experienced a decrease in hemoglobin levels of more than 1 mmol/L compared to their last known baseline value, all attributed to bleeding and none requiring transfusion. One patient in the pentaspline catheter group suffered a transient ischemic attack (TIA) postprocedure versus none in the circular catheter group (p = n.s.). This patient had a CHA_2_DS_2_‐VASc score of 1 and underwent a PVI‐only procedure. The patient experienced homonymous hemianopsia for 2 h on the third day postprocedure, without any detected abnormalities on the performed CT‐scan. Vagal reactions requiring temporal pacing and/or acute administration of (additional) atropine during ablation occurred in five patients (2.2%) that were treated with the pentaspline ablation catheter, compared to none of the patients that were treated with the circular catheter (p = n.s.)

Remarkably, one patient developed acute deep venous thrombosis in the right external iliac and common femoral veins approximately 2 weeks after treatment with the pentaspline ablation catheter, despite being on oral anticoagulation therapy. Furthermore, in one patient, silent cerebral ischemic lesions were detected on an MRI scan performed 4 days postprocedure, prompted by an altered mental status following ablation—presumably resulting from cerebral hypoperfusion in the context of pre‐existing cognitive impairment. No other adverse events occurred up to 30 days.

Several procedural characteristics were associated with the occurrence of adverse events and specifically bleeding events. The interactions between procedural characteristics and bleeding events are presented in Supporting Information Table 3.

## 4 Discussion

This manuscript describes the acute procedural outcomes of two commercially available PFA systems for PVI in a multicenter, real‐world clinical setting. This not only provides insight into the outcomes of PFA outside the well‐controlled setting of clinical trials, but also enables a direct comparison of two PFA systems.

Generally, this manuscript underlines the outcomes of pre‐commercial studies on PFA for PVI, as well as the outcomes from recent small comparative studies [16, 17]. Despite different procedural approaches in the centers included in this study, both PFA systems enabled successful isolation of all ablation targets, while keeping median procedure and fluoroscopy times as low as 41.0 and 10.0 min, respectively. This rate of acute isolation was similar to the outcomes of prior studies on PFA and thermal ablation systems, while total procedure and fluoroscopy times appear to be even shorter than the pre‐commercial clinical trials of PFA and most trials on thermal ablation systems [9, 11, 18, 19].

Direct comparison of the efficacy outcomes of both studied PFA systems revealed equal rates of acute isolation of the ablation targets. Likewise, the number of PVs that required more than the standard 8 energy applications and the total fluoroscopy duration were similar. However, there was a significant difference between the two studied PFA systems regarding the median total procedure duration, which was 36 min for the pentaspline catheter group and 49 min for the circular ablation catheter group. This difference might be explained by the differences in use of echocardiographic guidance, the use of electroanatomic mapping systems, and the use of conscious sedation (Supporting Information Table 1). Both procedural echocardiography (TEE or ICE) and electroanatomic mapping offer guidance to the operator, but require the manipulation of additional instruments during the procedure, which may increase the total procedure time. As both echocardiography and electroanatomic mapping were used significantly more often in the circular ablation catheter group, they may have contributed to the difference in total procedure time among the studied PFA systems. Additionally, conscious sedation was used significantly more often in the circular ablation catheter group, which also appears to result in longer procedure times. This could be due to patient movements that are not entirely suppressed during conscious sedation. However, it must be noted that the gains in procedure time of general anesthesia could be nullified by the time that is required for anesthesia induction, which was not recorded in the present study.

Major adverse events after catheter ablation for AF are rare. In this cohort, no patients experienced mortality, cardiac tamponade, or persistent phrenic nerve palsy. This aligns with prior studies on pulsed field and thermal ablation, which reported periprocedural rates for these complications of up to 0.3%, 1.3%, and 0.7%, respectively. The incidence of stroke and TIA was 0.7% of the entire cohort, which is slightly higher than rates in previous studies on pulsed field and thermal ablation [3, 9, 11, 20, 21]. Notably, the rate of stroke and TIA did not differ significantly between treatment arms. Lastly, vascular access complications requiring surgical intervention or transfusion were observed in 0.2% of cases, consistent with previous literature on this topic [3, 9, 11, 20, 21].

The rate of nonmajor adverse events in this study is mainly driven by vascular access site complications. Moreover, this cohort included no patients with transient phrenic nerve palsy, which is comparable to the low incidence rates found in literature [3, 9, 11, 20, 21]. However, nonmajor vascular access site complications were observed in as much as 7.2% of the cohort. This rate was higher than prior studies that reported rates of 1–4% [3, 20]. However, it must be noted that there is no standardized definition for minor bleeding complications, which may lead to underreporting in both our study and the existing literature, making it challenging to compare these outcomes effectively. Remarkably, in this cohort, nonmajor vascular complications occurred significantly more often in the pentaspline catheter group. This is likely due to the difference in outer sheath diameters between the pentaspline and circular catheter (16.8Fr *vs.* 14.0Fr, respectively).

While the application of ultrasound‐guided femoral puncture differed between groups, the use of closure devices (instead of a figure of eight suture and manual compression) was similar in both groups. Further assessment reveals that the application of ultrasound‐guided femoral puncture did not affect the rate of bleeding complications. However, the application of a closure device was associated with less vascular access site complications. This finding is in line with the recent STYLE‐AF randomized controlled trials that found a trend towards less minor vascular access‐related complications in patients treated with a vascular closure device [22]. Additionally, also other procedural characteristics seem to influence the number of vascular access‐site complications (Supporting Information table 3).

Finally, this study highlights some novel procedural aspects of PFA in everyday clinical practice, including the use of conscious sedation, ablation of non‐PV targets, and the use of electroanatomic mapping systems. Conscious sedation was used in 23.6% of the present cohort (10.6% of pentaspline and 40.6% of circular ablation group) and did not significantly affect procedural safety and efficacy. The widespread adoption of conscious sedation in this cohort was in contrast to prior studies on PFA in which general anesthesia was used for the vast majority of cases. A potential benefit of conscious sedation would be to obviate the additional risks of general anesthesia, as well cutting down on time and costs by averting the need for an anesthesiology team during the procedure. Targets outside the pulmonary veins were ablated in 13.7% of the cohort. Although prior trials did not show benefit of ablation outside the PVs for thermal ablation [23, 24]. The potentially enhanced efficacy of PFA could provide benefit in hard to ablate areas such as the posterior wall of the left atrium. Lastly, although both PFA systems are designed for fluoroscopy‐guided ablation of the PVs, electroanatomic mapping could be used to enable zero‐fluoroscopy ablation, to facilitate enhanced accuracy of ablation of non‐PV targets, and to improve ablation lesion assessment using voltage maps. The circular design of the PulseSelect™ catheter enables quick integration with current electroanatomic mapping systems, which is reflected in the current cohort (60 *vs.* 26%). Recently, the FARAVIEW™ software module, that enables visualization of the pentaspline ablation catheter, has gained FDA‐clearance. This could promote the use of electroanatomic mapping for the pentaspline‐based PFA system in future studies.

### 4.1 Limitations

This study is limited by its observational design, leading to missing data and potential underreporting of adverse events. However, per hospital protocol, all patients are closely monitored in the first hours following ablation, and most patients had at least one follow‐up visit at the operating center. We therefore believe that major adverse events are most likely captured in the electronic health record of the patients, which served as the primary data source for this study. The multicenter approach of this study could influence the outcomes of this study. Differences in the standard operating procedures, institutional learning curve, and variations in the included number of patients per treatment arm could contribute to this effect. Nonetheless, the authors believe that the multicenter design offers significant advantages over a single center study design by increasing the sample size and improving the generalizability of the outcomes. Although the selection of the PFA system was primarily based on system availability, selection bias cannot be completely excluded in this non‐randomized study, potentially limiting the comparability of outcomes. Finally, this manuscript reports only on the acute outcomes of the compared PFA systems. While recent studies found that the long‐term durability of PFA lesions is comparable to that of thermal ablation methods, and pre‐commercial studies reported similar arrhythmia recurrence rates, further research is necessary to evaluate the long‐term efficacy of the compared PFA systems [25, 26].

## 5 Conclusion

In conclusion, this is the first multicenter study comparing the efficacy and safety of the PulseSelect™ and FARAPULSE™ pulsed field ablation systems in a ‘real‐world’ setting. Our findings reveal significant differences between the ablation systems. However, both procedures demonstrated high procedural efficiency, great efficacy, and a low incidence of serious adverse events.

## Conflicts of Interest

The full disclosures of any potential conflict of interest are as follows: D.M. reports being a consultant for AltaThera Pharmaceuticals. B.K. has served as a consultant for and participated in PFA clinical trials sponsored by Abbott, Boston Scientific, and Medtronic. B.W. reports being a speaker/advisor for Medtronic and Boston Scientific. M.G. reports receiving speaker's honoraria, travel grants, and consulting honoraria from Abbott, Biosense Webster, Boston Scientific/Farapulse Inc, Medtronic, Lumavision, Biotronik, Bristol‐Myers Squibb, and Zoll. S.M. has served as a consultant for and participated in PFA clinical trials sponsored by Boston Scientific and Medtronic. V.H. received speaker's honoraria and consulting honoraria from Abbott, Biosense Webster, Boston Scientific, Medtronic, Zoll, and AltaThera Pharmaceuticals. L.B. reports being consultant/speaker/proctor for Boston Scientific and Medtronic and speaker for ZOLL, Biosense Webster and Abbott. All fees go to the cardiology department. The remaining authors declare no conflicts of int.

## Supporting information

Supporting Material revised.

## Data Availability

The data that support the findings of this study are available on request from the corresponding author. The data are not publicly available due to privacy or ethical restrictions.

## References

[1] J. P. Erinjeri and T. W. I. Clark , “Cryoablation: Mechanism of Action and Devices,” Journal of Vascular and Interventional Radiology 21, no. S8 (2010): S187–S191, 10.1016/j.jvir.2009.12.403. 20656228 PMC6661161

[2] D. Liu , Y. Li , and Q. Zhao , “Effects of Inflammatory Cell Death Caused by Catheter Ablation on Atrial Fibrillation,” Journal of Inflammation Research 16 (2023): 3491–3508, 10.2147/JIR.S422002. 37608882 PMC10441646

[3] E. Ekanem , V. Y. Reddy , B. Schmidt , et al., “Multi‐National Survey on the Methods, Efficacy, and Safety on the Post‐Approval Clinical use of Pulsed Field Ablation (MANIFEST‐PF),” EP Europace 24, no. 8 (2022): 1256–1266, 10.1093/europace/euac050. 35647644 PMC9435639

[4] T. Kotnik , L. Rems , M. Tarek , and D. Miklavčič , “Membrane Electroporation and Electropermeabilization: Mechanisms and Models,” Annual Review of Biophysics 48 (2019): 63–91, 10.1146/annurev-biophys-052118-115451. 30786231

[5] R. Cappato , H. Calkins , S.‐A. Chen , et al., “Updated Worldwide Survey on the Methods, Efficacy, and Safety of Catheter Ablation for Human Atrial Fibrillation,” Circulation: Arrhythmia and Electrophysiology 3, no. 1 (2010): 32–38, 10.1161/CIRCEP.109.859116. 19995881

[6] K.‐H. Kuck , J. Brugada , M. Schlüter , et al., “The FIRE and ICE Trial: What We Know, What We Can Still Learn, and What We Need to Address in the Future,” Journal of the American Heart Association 7, no. 24 (2018): e010777, 10.1161/JAHA.118.010777. 30561258 PMC6405614

[7] M. R. D. van de Kar , S. R. Slingerland , G. J. van Steenbergen , et al., “Pulsed Field Versus Cryoballoon Ablation for Atrial Fibrillation: A Real‐World Observational Study on Procedural Outcomes and Efficacy,” Netherlands Heart Journal 32, no. 4 (2024): 167–172, 10.1007/s12471-023-01850-8. 38291296 PMC10951164

[8] D. G. Della Rocca , L. Marcon , M. Magnocavallo , et al., “Pulsed Electric Field, Cryoballoon, and Radiofrequency for Paroxysmal Atrial Fibrillation Ablation: A Propensity Score‐Matched Comparison,” Europace 26, no. 1 (2023): euae016, 10.1093/europace/euae016. 38245007 PMC10823352

[9] A. Verma , D. E. Haines , L. V. Boersma , et al., “Pulsed Field Ablation for the Treatment of Atrial Fibrillation: PULSED AF Pivotal Trial,” Circulation 147, no. 19 (2023): 1422–1432, 10.1161/CIRCULATIONAHA.123.063988. 36877118 PMC10158608

[10] V. Y. Reddy , S. R. Dukkipati , P. Neuzil , et al., “Pulsed Field Ablation of Paroxysmal Atrial Fibrillation,” JACC: Clinical Electrophysiology 7, no. 5 (2021): 614–627, 10.1016/j.jacep.2021.02.014. 33933412

[11] V. Y. Reddy , E. P. Gerstenfeld , A. Natale , et al., “Pulsed Field or Conventional Thermal Ablation for Paroxysmal Atrial Fibrillation,” New England Journal of Medicine 389, no. 18 (2023): 1660–1671, 10.1056/NEJMoa2307291. 37634148

[12] E. Anter , M. Mansour , D. G. Nair , et al., “Dual‐Energy Lattice‐Tip Ablation System for Persistent Atrial Fibrillation: A Randomized Trial,” Nature Medicine 30, no. 8 (2024): 2303–2310, 10.1038/s41591-024-03022-6. PMC11333282 38760584

[13] M. K. Turagam , P. Neuzil , B. Schmidt , et al., “Safety and Effectiveness of Pulsed Field Ablation to Treat Atrial Fibrillation: One‐Year Outcomes From the MANIFEST‐PF Registry,” Circulation 148, no. 1 (2023): 35–46, 10.1161/CIRCULATIONAHA.123.064959. 37199171

[14] B. Schmidt , S. Bordignon , K. Neven , et al., “EUropean Real‐World Outcomes With Pulsed Field Ablation in Patients With Symptomatic Atrial Fibrillation: Lessons From the Multi‐Centre EU‐PORIA Registry,” Europace 25, no. 7 (2023): euad185, 10.1093/europace/euad185. 37379528 PMC10320231

[15] A. Metzner , M. Fiala , J. Vijgen , et al., “Long‐Term Outcomes of the Pentaspline Pulsed‐Field Ablation Catheter for the Treatment of Paroxysmal Atrial Fibrillation: Results of the Prospective, Multicentre Fara‐Freedom Study,” Europace 26, no. 3 (2024): euae053, 10.1093/europace/euae053. 38385529 PMC10932745

[16] L. Katov , Y. Teumer , C. Bothner , W. Rottbauer , and K. Weinmann‐Emhardt , “Comparative Analysis of Real‐World Clinical Outcomes of a Novel Pulsed Field Ablation System for Pulmonary Vein Isolation: The Prospective CIRCLE‐PVI Study,” Journal of Clinical Medicine 13, no. 23 (2024): 7040, 10.3390/jcm13237040. 39685499 PMC11642190

[17] M. M. Zylla , C. Mages , A.‐K. Rahm , et al., “Comparative Evaluation of 2 Pulsed Field Ablation Systems for Atrial Fibrillation: Insights From Real‐World Clinical Implementation and Short‐Term Outcomes,” Heart Rhythm: The Official Journal of the Heart Rhythm Society 1 (November 2024): 1, 10.1016/j.hrthm.2024.10.068. 39515496

[18] J. G. Andrade , J. Champagne , M. Dubuc , et al., “Cryoballoon or Radiofrequency Ablation for Atrial Fibrillation Assessed by Continuous Monitoring: A Randomized Clinical Trial,” Circulation 140, no. 22 (2019): 1779–1788, 10.1161/CIRCULATIONAHA.119.042622. 31630538

[19] K.‐H. Kuck , J. Brugada , A. Fürnkranz , et al., “Cryoballoon or Radiofrequency Ablation for Paroxysmal Atrial Fibrillation,” New England Journal of Medicine 374, no. 23 (2016): 2235–2245, 10.1056/NEJMoa1602014. 27042964

[20] S. Tzeis , E. P. Gerstenfeld , J. Kalman , et al., “2024 European Heart Rhythm Association/Heart Rhythm Society/Asia Pacific Heart Rhythm Society/Latin American Heart Rhythm Society Expert Consensus Statement on Catheter and Surgical Ablation of Atrial Fibrillation,” Europace: European Pacing, Arrhythmias, and Cardiac Electrophysiology: Journal of the Working Groups on Cardiac Pacing, Arrhythmias, and Cardiac Cellular Electrophysiology of the European Society of Cardiology 26, no. 4 (2024): euae043, 10.1093/europace/euae043. 38587017 PMC11000153

[21] K. Benali , P. Khairy , N. Hammache , et al., “Procedure‐Related Complications of Catheter Ablation for Atrial Fibrillation,” Journal of the American College of Cardiology 81, no. 21 (2023): 2089–2099, 10.1016/j.jacc.2023.03.418. 37225362

[22] R. R. Tilz , M. Feher , J. Vogler , et al., “Venous Vascular Closure System vs. Figure‐of‐Eight Suture Following Atrial Fibrillation Ablation: The STYLE‐AF Study,” Europace: European Pacing, Arrhythmias, and Cardiac Electrophysiology: Journal of the Working Groups on Cardiac Pacing, Arrhythmias, and Cardiac Cellular Electrophysiology of the European Society of Cardiology 26, no. 5 (2024): euae105, 10.1093/europace/euae105. 38647070 PMC11210072

[23] A. Verma , C. Jiang , T. R. Betts , et al., “Approaches to Catheter Ablation for Persistent Atrial Fibrillation,” New England Journal of Medicine 372, no. 19 (2015): 1812–1822, 10.1056/NEJMoa1408288. 25946280

[24] P. M. Kistler , D. Chieng , H. Sugumar , et al., “Effect of Catheter Ablation Using Pulmonary Vein Isolation With Vs Without Posterior Left Atrial Wall Isolation on Atrial Arrhythmia Recurrence in Patients With Persistent Atrial Fibrillation: The CAPLA Randomized Clinical Trial,” Journal of the American Medical Association 329, no. 2 (2023): 127–135, 10.1001/jama.2022.23722. 36625809 PMC9856612

[25] S. Tohoku , K. R. J. Chun , S. Bordignon , et al., “Findings From Repeat Ablation Using High‐Density Mapping After Pulmonary Vein Isolation With Pulsed Field Ablation,” EP Europace 25, no. 2 (2023): 433–440, 10.1093/europace/euac211. PMC9935020 36427201

[26] M. A. Gunawardene , G. Frommeyer , C. Ellermann , et al., “Left Atrial Posterior Wall Isolation With Pulsed Field Ablation in Persistent Atrial Fibrillation,” Journal of Clinical Medicine 12, no. 19 (2023): 6304, 10.3390/jcm12196304. 37834948 PMC10573684

