# Supplementary material for: Direct Comparison of Two Commercially Available Pulsed Field Ablation Systems for Atrial Fibrillation; Procedure Characteristics and Acute Outcomes
Source: J Cardiovasc Electrophysiol. 2025 Jun 12;36(8):1957–65. doi: 10.1111/jce.16761 (PMC12337626; doi:10.1111/jce.16761)
Supplement: Supplementary file 1 — Supporting Material revised. [file JCE-36-1957-s001.docx]

**Supplementary Table 1.** Procedure times, stratified by procedural characteristics

|  | No | Yes | p-value |
| --- | --- | --- | --- |
| General anesthesia | 50.0 [44.5; 66.0] | 38.0 [32.0; 51.0] | <0.001 |
| Ultrasound guided vascular access | 40.5 [36.0; 46.8] | 41.0 [33.0; 60.0] | 0.444 |
| Periprocedural use of TEE | 40.0 [33.0; 58.0] | 47.0 [42.5; 49.0] | 0.113 |
| ICE-guided procedure | 39.0 [33.0; 46.0] | 77.0 [ 45.2; 126.8] | <0.001 |
| Electroanatomic mapping system | 36.0 [32.0; 43.0] | 63.0 [45.5; 111.5] | <0.001 |
| Lesions beyond PVI | 39.0 [33.0; 50.0] | 112.0 [56.3; 141.3] | <0.001 |
| Vascular closure device | 40.0 [33.0; 50.0] | 57.0 [35.3; 87.8] | <0.001 |
| Protamine | 44.0 [37.5; 60.0] | 36.0 [30.4; 48.5] | <0.001 |

Numbers in the cells are median procedure time in minutes and [interquartile range]. The p-value was calculated using the Kruskal-Wallis test.

**Supplementary Table 2.** Fluoroscopy times, stratified by procedural characteristics

|  | No | Yes | p-value |
| --- | --- | --- | --- |
| General anesthesia | 8.7 [5.0; 12.0] | 10.0 [8.0; 15.0] | <0.001 |
| Ultrasound guided vascular access | 11.0 [9.0; 14.0] | 10.0 [7.0; 14.2] | 0.011 |
| Periprocedural use of TEE | 10.0 [8.0; 15.0] | 5.0 [3.5; 8.0] | <0.001 |
| ICE-guided procedure | 10.0 [8.0; 13.0] | 9.5 [0.0; 20.1] | 0.278 |
| Electroanatomic mapping system | 10.0 [8.0; 13.0] | 8.1 [3.0; 18.1] | 0.007 |
| Lesions beyond PVI | 10.0 [7.0; 13.0] | 16.0 [7.9; 24.5] | <0.001 |

Numbers in the cells are median fluoroscopy time in minutes and [interquartile range]. The p-value was calculated using the Kruskal-Wallis test.

**Supplementary Table 3.** Number of bleeding events, stratified by procedural characteristics

|  | No | Yes | p-value |
| --- | --- | --- | --- |
| General anesthesia | 0/95 (0.0%) | 30/307 (9.8%) | 0.003 |
| Ultrasound guided vascular access | 3/54 (5.6%) | 27/348 (7.8%) | 0.768 |
| Periprocedural use of TEE | 29/375 (7.7%) | 1/27 (3.7%) | 0.696 |
| ICE-guided procedure | 30/288 (10.4%) | 0/114 (0.0%) | 0.001 |
| Electroanatomic mapping system | 28/238 (11.8%) | 2/164 (1.2%) | <0.001 |
| Lesions beyond PVI | 30/347 (8.6%) | 0/55 (0.0%) | 0.047 |
| Vascular closure device | 30/311 (9.6%) | 0/91 (0.0%) | 0.004 |
| Protamine | 17/269 (6.3%) | 13/133 (9.8%) | 0.299 |

Numbers in the cells are the number of cases with a bleeding event / total number of cases, with corresponding percentage. The p-value was calculated using the Fisher's exact test.
